# Tablet Compression and Performance of *Artocarpus altilis* as a Starch-Rich Natural-Source Excipient with Different APIs

**DOI:** 10.3390/pharmaceutics18070819

**Published:** 2026-07-02

**Authors:** Luis F. Torrens-Sotomayor, Carlos Velázquez-Figueroa

**Affiliations:** Center for Pharmaceutical Engineering Development and Learning, Department of Chemical Engineering, University of Puerto Rico at Mayagüez, Call Box 9000, Mayagüez, PR 00681-9000, USA; luis.torrens@upr.edu

**Keywords:** tablet compression, starch-based excipient, *Artocarpus altilis*, tabletability, compactability, compressibility, biopharmaceutics classification system, dissolution performance

## Abstract

**Background/Objectives**: Excipients play a key role in the manufacturability and performance of solid oral dosage forms, particularly for drug candidates with diverse solubility, permeability, and mechanical properties. Understanding excipient behavior during tablet compression is essential for robust formulation design. Starch-rich natural-source material have emerged as promising candidates due to their availability and favorable deformation behavior. However, there is limited understanding of their tablet compression performance and interactions with active pharmaceutical ingredients (APIs). This study aimed to evaluate the tablet compression behavior and functional performance of a starch-rich natural-source excipient prepared from whole *Artocarpus altilis* fruit material when formulated with APIs representing all four classes of the Biopharmaceutics Classification System. **Methods**: Tablets were prepared using a hydraulic press at compression pressures ranging between 296 and 591 MPa. Model APIs included acetaminophen, clarithromycin, vitamin C, and berberine hydrochloride. Tablet compression behavior was characterized using tabletability, compressibility, and compactability profiles, while tablet performance was evaluated through friability, disintegration, and dissolution testing. **Results**: Densification behavior was controlled primarily by the *Artocarpus altilis* excipient, whereas tensile strength development and compactability were strongly influenced by API properties. Formulations containing clarithromycin and berberine HCl exhibited enhanced tabletability and compactability, achieving higher tensile strengths at comparable solid fractions. Acetaminophen and vitamin C formulations showed limited strength gains despite similar densification. Formulations containing clarithromycin and berberine HCl maintained low friability, while enabling rapid disintegration and dissolution in acetaminophen and vitamin C formulations. **Conclusions**: Tabletability and compactability trends varied among formulations, likely reflecting differences in API physical properties and formulation-dependent interparticle interactions, whereas compressibility primarily reflected excipient-controlled densification. Distinct trends were observed across BCS classes, with low-solubility APIs producing stronger tablets and highly soluble APIs showing lower mechanical strength and faster disintegration. Overall, *Artocarpus altilis* functions as a mechanically robust yet performance-adaptive excipient suitable for tablet formulations across diverse biopharmaceutical contexts.

## 1. Introduction

In solid oral dosage forms, therapeutic performance depends on the combined behavior of the active pharmaceutical ingredient (API) and the excipients under both processing and physiological conditions. Excipients play a critical role beyond serving as inert fillers, as they directly influence formulation properties such as powder flow, compaction behavior, mechanical integrity, disintegration, dissolution, and drug availability [1,2,3]. This role makes the selection and design of modern excipients critical to the development of robust solid oral formulations, particularly as modern drug candidates increasingly present challenges related to solubility, permeability, and mechanical behavior [4,5,6,7].

Many commercially available excipients exhibit limited functional flexibility and often require extensive chemical modification or co-processing to meet formulation requirements [8,9,10]. These approaches can increase manufacturing complexity and restrict the formulation design space. In response, regulatory agencies such as the U.S. Food and Drug Administration (FDA) have emphasized the need for novel excipients capable of expanding functionality while maintaining manufacturing feasibility and regulatory compliance [11]. Additionally, the development of such excipients requires a fundamental understanding of their behavior during critical unit operations, particularly tablet compression, where material properties and process conditions interact to determine final product performance.

Given the need to enhance excipient functionality while maintaining manufacturability, starch-based materials have been extensively studied as pharmaceutical excipients due to their availability, biocompatibility, and favorable deformation behavior during compaction [12,13]. Previous studies have evaluated starches derived from various botanical sources, primarily focusing on their use as binders or disintegrants, and often relying on extraction or modification processes to tailor their performance [14,15,16,17,18,19,20].

Recent studies have analyzed the use of whole food-derived ingredients rather than isolated components for tablet formation [21,22,23,24]. Among these materials, *Artocarpus altilis* has emerged as a promising natural-source, starch-rich excipient with potential pharmaceutical applications [25,26]. Previous work has demonstrated the feasibility of using chemically unmodified breadfruit granules as excipients, showing acceptable tablet formation and performance [27]. However, limited studies have examined the use of whole food matrices as excipients and their interactions with APIs of differing physicochemical characteristics, as well as how these interactions influence compression behavior and final tablet performance.

This study focuses on evaluating the tablet compression behavior and performance attributes of *Artocarpus altilis* as a natural-source, high-starch excipient formulated with different APIs. These APIs were selected using the Biopharmaceutics Classification System (BCS), which provides a framework to categorize APIs based on solubility and permeability [28], including acetaminophen (BCS Class I), clarithromycin (BCS Class II), vitamin C (BCS Class III), and berberine HCl (BCS Class IV). Tablet compression behavior (i.e., tabletability, compressibility, and compactability profiles) was examined following the United States Pharmacopeia (USP) protocol USP <1062>, together with critical quality attributes (i.e., friability, disintegration, and dissolution behavior) to understand the relationship between API-excipient interactions and tablet performance. By understanding these formulation behaviors, this work aims to provide mechanistic insight into API-excipient interactions by systematically evaluating the compression behavior of a starch-rich natural-source excipient derived from whole *Artocarpus altilis* across APIs representing all four BCS classes. These findings may also support the development of sustainable and environmentally conscious formulation strategies based on non-toxic, natural-source excipients for modern solid oral dosage forms.

## 2. Materials and Methods

### 2.1. Natural-Source High-Starch Excipient

For the natural-source, starch-rich excipient, whole *Artocarpus altilis* (white cultivar) was used. The raw material was harvested in Puerto Rico and sourced primarily from the southwest region of the island. The processing workflow followed the procedure previously reported by Torrens-Sotomayor et al. [27], with minor adjustments specific to the present study. No starch extraction or additional chemical or physical modification was performed. The material was used as a whole-food-derived powder to evaluate its performance as a natural-source excipient.

The material was dried in a Heratherm^TM^ oven (Thermo Fisher Scientific, Waltham, MA, USA) at 55 °C until a target moisture content of approximately 10 wt.% was reached. The measured moisture content of the processed powder was 10.97 ± 0.33%, determined according to AOAC Method 925.10 for moisture determination [29]. The dried material was milled using a FitzMill Comminutor Model L1A (The Fitzpatrick Company, Elmhurst, IL, USA) at 3000 rpm and a 685.8 µm round-hole perforated screen.

The physicochemical properties of the resulting powder were characterized to evaluate its suitability as a pharmaceutical excipient. Particle size distribution was determined using laser diffraction (Insitec RT Sizer, Malvern Panalytical Ltd., Malvern, UK).The total starch content of the material was determined using the Total Starch Assay Protocol (K-TSTA-50A/K-TSTA-100A; Megazyme, Bray, Ireland) following AOAC Method 996.11 and AACC Method 76-13.01.The solubility of the powder was determined using the water solubility index following a method based on Anderson et al., as described in previous studies [30]. The aqueous solubility of the powder was evaluated using the water solubility index (WSI) following a method based on Anderson et al., as described in previous studies [30]. Measurements were conducted at 21 °C and 37 °C to assess the temperature dependence of solubility.

### 2.2. Active Pharmaceutical Ingredients

**Figure A1:**
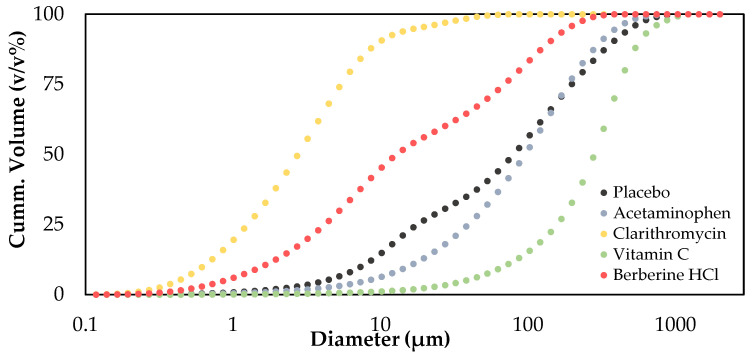
Cumulative volumetric particle size distributions of placebo and API powders used in this study, including acetaminophen, clarithromycin, vitamin C, and berberine HCl.

APIs were selected using the four classes of the Biopharmaceutics Classification System (BCS), allowing the evaluation of APIs with distinct solubility and permeability characteristics and their potential interactions with the excipient. For all formulations, APIs were physically blended with the excipient without any chemical modification or addition of synthetic binders or disintegrants. The concentration of each API was selected based on typical therapeutic dose ranges and the physicochemical properties of each drug. The cumulative particle size distributions of the excipient and API powders are shown in Figure A1 (Appendix A).

Acetaminophen (CAS No. 103-90-2, MW: 151.16 g/mol) was supplied by Hebei Jiheng Pharmaceutical Co., Ltd. (Hengshui, China; lot no. 1109041) and used as supplied without further purification. Acetaminophen is commonly classified as BCS Class I compound due to its high aqueous solubility and high permeability [31,32]. The particles exhibited predominantly irregular, angular to polyhedral morphology, with well-defined edges and relatively smooth, faceted surfaces. The median particle size (D50) of the acetaminophen powder was 95.46 µm. Tablets were formulated containing 500 mg of acetaminophen per tablet. Commercial acetaminophen tablets (500 mg, Panadol^®^, GSK Consumer Healthcare, Warren, NJ, USA) were used as a reference formulation for dissolution and performance comparisons.

Clarithromycin (CAS No. 81103-11-9, MW: 747.95 g/mol) was supplied as a commercial batch (Lot No. EX000013–400112) (Mutchler Inc., Bayamón, PR, USA) and used as received. Clarithromycin is classified as a BCS Class II compound due to its low aqueous solubility and high permeability [31]. The particles exhibited a fine particle size (D50 = 2.76 µm) and an irregular morphology with a tendency towards agglomeration. Experimental tablets contained 250 mg of clarithromycin per tablet. Commercial clarithromycin tablets (250 mg, Clarithromycin Tablets, USP; Rising Health, LLC, Saddle Brook, NJ, USA) were used as a reference formulation for dissolution and tablet performance comparisons.

L-ascorbic acid (vitamin C, CAS No. 50-81-7, MW: 176.12 g/mol) was obtained from Nutricost (Ascorbic Acid; Nutricost, Vineyard, UT, USA) and used as supplied without further purification. Ascorbic acid is classified as BCS Class III compound due to its high aqueous solubility and low permeability [33,34]. The particles exhibited well-defined crystalline morphology with sharp edges and a relatively large particle size (D50 = 281.25 µm). Tablets were formulated with 250 mg of vitamin C per tablet.

Berberine hydrochloride (CAS No. 633-65-8, MW: 371.81 g/mol) was obtained as a powder from BulkSupplements (Berberine HCl; BulkSupplements, Henderson, NV, USA) and used as received. The particles exhibited tabular to plate-like morphology, with a D50 of 12.93 µm. Berberine hydrochloride is frequently reported as a poorly soluble and poorly permeable compound, often described as consistent with BCS Class IV behavior, although classification may vary depending on the source and experimental criteria reported in the literature [35]. Tablets were formulated with 500 mg of berberine HCl per tablet.

### 2.3. Tablet Compression Procedure

The tablet densification was performed using a Carver Hydraulic Press Model 3912 (Carver, Inc., Wabash, IN, USA). Compression was conducted under single-sided loading conditions using flat-faced cylindrical punches made of 440 stainless steel and a nominal diameter of 13 mm, a fixed tablet mass of 1.0 g, and a constant dwell time. Compression pressure (MPa) was calculated from the applied force recorded by the hydraulic press gauge and normalized by the nominal punch cross-sectional area. No external lubrication was applied to the die or punches to isolate the API–excipient compaction behavior.

A total of five compression forces corresponding to compression pressures of 296, 369, 443, 517, and 591 MPa were analyzed. This pressure range was selected based on previous work with the same material system [27], where tablet formation was observed within this range when using whole-material *A. altilis* powders without external excipients or functional modifiers (e.g., binders) incorporated to the formulation.

### 2.4. Tablet Compression Characterization

Tablet compression behavior was characterized following the general framework described in USP 〈1062〉. Tablet tensile strength (*σₜ*) was calculated from the diametral compression test for flat-faced cylindrical tablets using Equation (1), where *F* is the tablet breaking force, *D* is the tablet diameter, and *h* is the tablet thickness measured using a digital caliper (Husky, Milton, VT, USA). Tablet *solid fraction* was calculated according to Equation (2), where *m* is the tablet mass, *ρ_p_* is the true particle density, and *V* is the tablet volume.

(1) $$\sigma_{t}=\frac{2F}{\pi Dh}$$

(2) $$\mathrm{solid}\;\mathrm{fraction}=\frac{m}{\rho_{p}V}$$

Tabletability was evaluated by relating tablet tensile strength to applied compression pressure using the log–log relationship shown in Equation (3), where *K* represents the tabletability coefficient and *B* represents the extrapolated tensile strength at unit compression pressure [36,37]. Compressibility was evaluated by relating compression pressure to tablet solid fraction using the semi-logarithmic relationship shown in Equation (4), where *a* describes the sensitivity of pressure to changes in solid fraction and *b* represents the pressure required to initiate densification [38]. Compactability was evaluated by relating tablet tensile strength directly to tablet solid fraction using Equation (5), known as the Ryshkewitch–Duckworth equation, where *k* represents the compactability coefficient and *A* represents the extrapolated tensile strength at zero solid fraction [39].

(3) $$\log\left(\sigma_{t}\right)=K\times log(P)+B$$

(4) $$\log\left(P\right)=a\times (\mathrm{solid}\;\mathrm{fraction})+b$$

(5) $$\log\left(\sigma_{t}\right)=k\times (\mathrm{solid}\;\mathrm{fraction})+A$$

### 2.5. Tablet Performance Characterization

Tablet friability was evaluated using a Vanderkamp 10809 Model (VanKel Technology Group, Cary, NC, USA) following the procedure described in USP 〈1216〉. Tablets were rotated at 25 rpm for 4 min, after which friability was expressed as the percentage weight loss relative to the initial tablet mass. Tablet disintegration testing was performed according to USP 〈701〉 for uncoated tablets using distilled water maintained at 37 ± 0.5 °C.

Dissolution testing was conducted using the SR8 Plus Dissolution Test Station, Auto Plus Maximizer, and Auto Plus MultiFill from Hanson Research (Chatsworth, CA, USA), following USP 〈711〉 with slight modifications. The equipment setup was based on the USP Apparatus 2 (paddles) configuration for immediate-release tablets, as specified in the USP guidelines. Paddles were used as the agitation method at 50 rpm, at a height of 25 ± 2 mm from the bottom of the vessel. The dissolution medium consisted of 900 mL of distilled water (pH = 6.28 ± 0.39) and was maintained at 37 ± 0.5 °C. Samples were withdrawn using a 70 µm polyethylene filter tip (1/8 in, Hanson Research) at 0, 1, 3, 5, 7, 9, 10, 12, 15, 20, 30, 45, and 60 min, and analyzed using a Genesys 10S UV-Vis spectrophotometer (ThermoScientific, Waltham, MA, USA). Dissolution results were reported as relative solubility, defined as the ratio between the measured dissolved amount at a given time and the maximum solubility of the API under the test conditions.

The area under the dissolution curve (AUC) was used as a measure of cumulative drug release over time and was calculated by numerical integration of the relative solubility–time profile up to the final sampling time. Dissolution efficiency (DE) was calculated as the ratio of the area under the dissolution curve (AUC) up to 60 min to the area od a rectangle corresponding to the complete drug dissolution over the same time interval. Characteristic dissolution times (t_10_, t_50_, and t_80_), were determined directly from the dissolution profiles. Results of these values are presented in Figure A3 (Appendix C).

To describe dissolution kinetics, experimental dissolution data were fitted to several commonly used models, including the zero-order, first-order, Higuchi, Hixson–Crowell, Korsmeyer–Peppas, and Weibull models. Among the evaluated models, the simplified Weibull model (Equation (6)) provided the best overall fit to the dissolution profiles across all formulations and compression pressures, where *F*(*t*) is the fraction of drug dissolved at time *t*, α is the scale parameter related to the characteristic dissolution time, and *β* is the shape parameter that describes the curvature of the dissolution profile [40].

(6) $$F\left(t\right)=1-\exp\left[-{\left(\frac{t}{\alpha }\right)}^{\beta }\right]$$

### 2.6. Experimental Design

The experimental design evaluated the combined effects of API physicochemical properties and compression conditions on tablet compression and performance attributes. The APIs studied included acetaminophen (BCS Class I), clarithromycin (BCS Class II), vitamin C (BCS Class III), and berberine HCl (BCS Class IV). Tablets were prepared at a fixed total weight of 1.0 g, with API loadings corresponding to typical therapeutic strengths. Acetaminophen and berberine HCl were incorporated at 50% *w*/*w* (500 mg per tablet), whereas clarithromycin and vitamin C were formulated at 25% *w*/*w* (250 mg per tablet), with the remaining fraction composed of *Artocarpus altilis* as the excipient. Tablet compaction was performed at 4, 5, 6, 7, and 8 metric tons, corresponding to 296, 369, 443, 517, and 591 MPa, respectively. Tablet mass, tooling geometry, dwell time, and loading conditions were maintained constant throughout the study.

To assess statistical differences among tablet performance attributes, a one-way ANOVA was conducted followed by Tukey’s post hoc test, both at a 95% confidence level, using Minitab^®^ 21 (version 21.1). Dissolution data were fitted to multiple kinetic models, and model selection was based on the coefficient of determination (*R*^2^), sum of squared errors (SSE), and mean absolute residuals (MAR). Placebo tablets composed solely of the natural-source excipient were used as negative controls. Commercial tablet formulations were used as controls for friability, disintegration, and dissolution testing, and their performance metrics are summarized in Appendix B. All experiments were performed in 4–6 replicates per condition (n = 4–6). Tablets made only with *A. altilis* were designated as placebo tablets and were used as the negative control.

## 3. Results

### 3.1. Physicochemical Characterization of the Artocarpus altilis Excipient

The resulting *Artocarpus altilis* powder had a volume-based median particle size (D50) of 79.84 μm. Powder flow behavior, morphology, and density for this excipient have been previously reported by Torrens-Sotomayor et al. [27]. According to the classification described in USP 〈1174〉 Powder Flow, the material exhibited poor flow characteristics, which is consistent with its irregular and angular, cube-like morphology. True particle density for this powder was reported to be 1.60 ± 0.09 g/cm^3^.

This excipient exhibited a high starch content of 63.49 ± 0.67%, confirming its starch-rich composition and aligning with values reported in the literature for *A. altilis* [40,41]. This powder also exhibited low aqueous solubility, with values of 9.77 ± 0.58% at 21 °C and 10.03 ± 0.75% at 37 °C, indicating that the material is largely water-insoluble, with only a small fraction of soluble components released in water. Despite this limited solubility, the starch-rich composition provides hydrophilic functional groups capable of interacting with water, which is consistent with the typical behavior of starch-based materials [41].

### 3.2. Tablet Compression Profile

**Figure 1:**
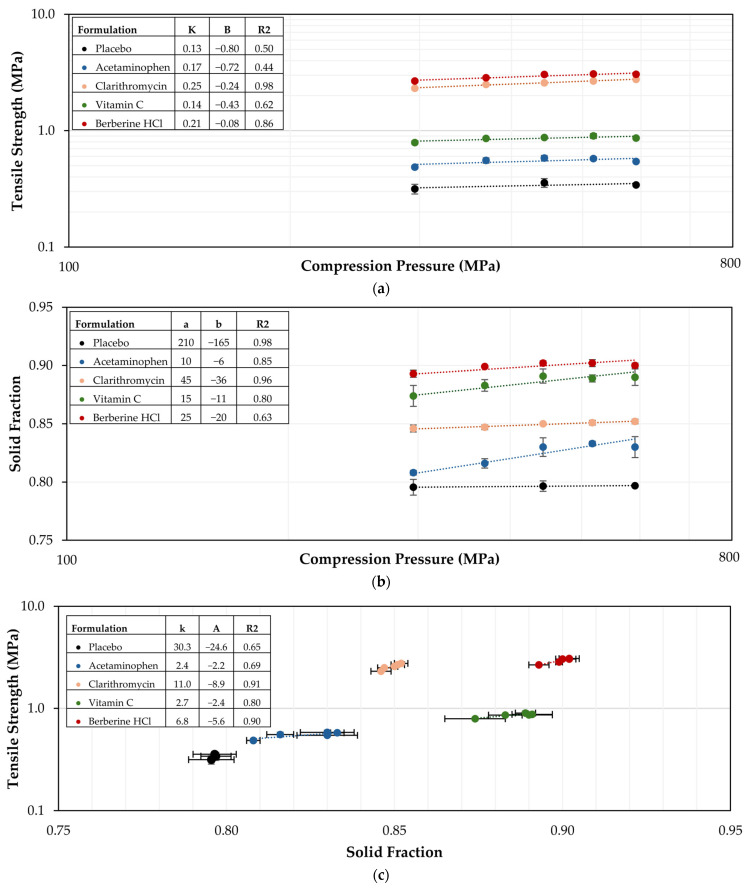
Tablet compression profiles of *Artocarpus altilis*-based formulations containing APIs across different BCS classes, including acetaminophen, clarithromycin, vitamin C, and berberine HCl. Compression profile includes (**a**) tabletability, (**b**) compressibility, and (**c**) compactability. Plots include descriptive model fits and corresponding parameters according to USP <1062>. (n = 6).

Figure 1 presents the tablet compression profile of *Artocarpus altilis*-based formulations containing drugs representing different BCS classes, evaluated in terms of tabletability, compressibility, and compactability. Results indicate that while the excipient establishes consistent densification behavior, API properties significantly influence mechanical strength development, especially tensile strength.

The tabletability profile (Figure 1a) showed that all formulations exhibited increasing tensile strength with increasing compression pressure, consistent with typical powder densification behavior. Formulations containing clarithromycin and berberine HCl reached tensile strengths of approximately 2.7–3.1 MPa, compared to 0.32–0.36 MPa for the placebo, representing an increase of approximately 8–9-fold relative to the placebo. Clarithromycin and berberine HCl exhibited fine particle size distributions, which may increase the number of contact points within the excipient matrix and promote efficient API–excipient bond formation, thereby contributing to higher tensile strength.

Acetaminophen and vitamin C exhibited more limited increases in tensile strength, reaching approximately 0.55–0.58 MPa and 0.86–0.90 MPa, respectively. These values correspond to approximately 1.6–1.8-fold for acetaminophen and 2.5–2.8-fold for vitamin C increases relative to the placebo. Acetaminophen and vitamin C exhibited larger median particle sizes compared to the other APIs, which may have reduced contact point density at high API loading, thereby limiting bond formation and tensile strength development.

Evaluating the tabletability constants, it was observed that formulations containing low solubility APIs (i.e., clarithromycin and berberine HCl) exhibited higher K values, indicating a greater intrinsic ability to develop tensile strength under compression. This behavior may be associated with differences in particle size distribution, morphology, and deformation behavior, which can promote more effective API–excipient interactions and enhance interparticle contact and bonding during compression. In contrast, acetaminophen and vitamin C may exhibit reduced interparticle contact efficiency, resulting in lower strength gains despite similar densification.

The compressibility profile (Figure 1b) showed that all formulations exhibited a gradual increase in solid fraction with increasing compression pressure, following pressure-dependent densification trends. Acetaminophen exhibited a more pronounced pressure-dependent increase in solid fraction compared to the other formulations, indicating greater sensitivity of densification to compression pressure. This behavior may be partially attributed to the elongated, irregular crystalline shape of acetaminophen, which may enhance particle rearrangement and packing efficiency under load. However, this enhanced densification did not result in a corresponding increase in tensile strength, demonstrating that differences in tabletability do not solely depend on compressibility but also on morphology- and particle size-driven differences in contact density and bonding efficiency. This general trend is consistent with the behavior of starch-rich materials, where densification occurs mainly at lower pressures, with smaller incremental changes at higher pressures [42,43,44].

The fitted compressibility parameters indicate that API incorporation results in only minor shifts in the solid fraction intercept, while the overall pressure-dependent densification trend remains unchanged. This suggests that densification depends primarily on the *A. altilis* excipient matrix. Across formulations, solid fractions approached values commonly reported for compressed pharmaceutical tablets (≈0.85). However, since large differences in tensile strength were observed at comparable pressures, mechanical performance is not solely governed by compressibility. This distinction indicates that compressibility reflects excipient-controlled densification, whereas differences in tabletability and compactability arise from formulation-dependent effects associated with API particle properties and their influence on interparticle bonding.

The compactability profile (Figure 1c) showed distinct strength–density regions across formulations. The placebo and acetaminophen tablets cluster in a low-strength region (≤0.6 MPa) despite reaching solid fractions of approximately 0.80–0.83, indicating limited efficiency in translating densification into mechanical strength. Vitamin C was located in an intermediate region, achieving tensile strengths approaching 0.9 MPa at solid fractions near 0.88–0.89. In contrast, formulations containing clarithromycin and berberine HCl were in a high-strength regime, reaching tensile strengths above 2.5 MPa at solid fractions of ~0.85–0.90. This behavior is consistent with higher effective contact density and improved interparticle bonding within the compact.

These distinct regions indicate that formulations containing clarithromycin and berberine HCl exhibit a more efficient conversion of densification into tensile strength, whereas acetaminophen and vitamin C formulations show more moderate strength gains. This trend is supported by the compactability constants, where clarithromycin and berberine HCl display higher k values and R^2^ values, indicating a more efficient relationship between solid fraction and tensile strength.

### 3.3. Tablet Performance Attributes

#### 3.3.1. Friability

**Figure 2:**
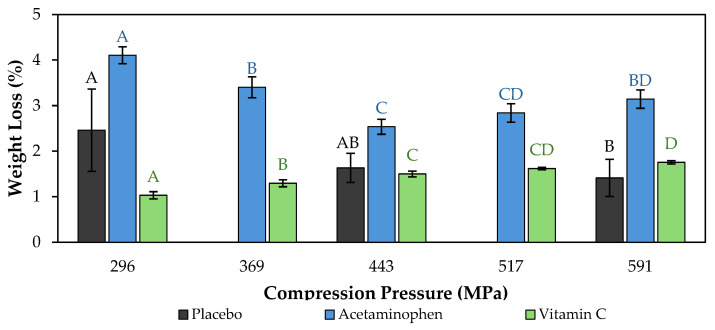
Effect of compression pressure on tablet friability of high-solubility API formulations (acetaminophen (BCS Class I) and vitamin C (BCS Class III)) and placebo prepared with *Artocarpus altilis* as the excipient. Statistical comparisons were performed across compression pressures within each formulation using one-way ANOVA and Tukey’s post hoc test (95% confidence level). Different letters indicate significant differences (*p* < 0.05). (n = 4–6).

Tablet friability was influenced by both compression pressure and API properties, decreasing or remaining constant with increasing compression pressure, consistent with enhanced particle–particle bonding and reduced porosity at higher compaction stresses. For highly soluble APIs, distinct pressure-dependent trends were observed, as depicted in Figure 2, showing the effect of API physical properties, particle morphology, and compaction behavior.

Acetaminophen showed high friability values ranging from 2.54 to 4.11%, corresponding to approximately 1.7–2.0-fold higher friability than the placebo across all compression pressures (*p* < 0.0001). The U-shaped trend suggests a transition from densification-dominated behavior to a regime governed by brittle fracture and limited plastic deformation, as previously reported [45,46].

Vitamin C tablets exhibited an increase in friability with increasing compression pressure, from 1.03 ± 0.08% at 296 MPa to 1.75 ± 0.04% at 591 MPa. This pressure-dependent increase indicates that the mechanical properties of vitamin C limit effective interparticle bonding during compaction. Ascorbic acid is known to exhibit brittle fragmentation behavior associated with its crystal packing arrangement and strong intermolecular hydrogen bonding, which restrict the formation of extensive bonding areas during compression [47]. Under higher compaction stresses, this brittle nature may promote fragmentation without sufficient bonding efficiency, resulting in weaker tablet structures and increased friability.

**Figure 3:**
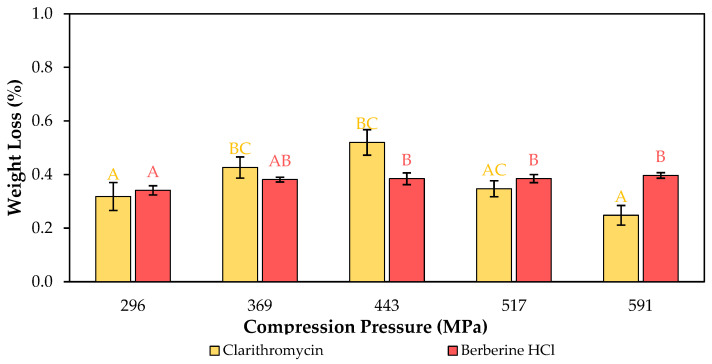
Effect of compression pressure on tablet friability of low-solubility API formulations (clarithromycin (BCS Class II) and berberine HCl (BCS Class IV)) prepared with *Artocarpus altilis* as the excipient. Statistical comparisons were performed across compression pressures within each formulation using one-way ANOVA and Tukey’s post hoc test (95% confidence level). Different letters indicate significant differences (*p* < 0.05). (n = 4–6).

Low solubility formulations exhibited lower friability, with values remaining < 0.55% across all compression pressures, as shown in Figure 3. Clarithromycin tablets showed friability values between 0.25 and 0.52%, while berberine HCl tablets remained similar across compression pressures ranging from 0.34 to 0.40%. The low friability observed for both drugs can be attributed to their finer particle size distribution, which may limit moisture-induced weakening and promotes a higher density of contact points within the starch matrix, thus improving tablet mechanical stability [48,49].

#### 3.3.2. Disintegration

**Figure 4:**
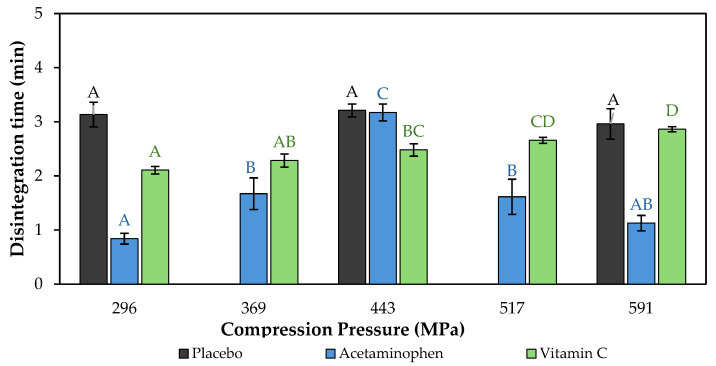
Effect of compression pressure on disintegration time for placebo and API-containing high solubility formulations (acetaminophen (BCS Class I), and vitamin C (BCS Class III)) using *Artocarpus altilis* as the excipient. Statistical comparisons were performed across compression pressures within each formulation using one-way ANOVA and Tukey’s post hoc test (95% confidence level). Different letters indicate significant differences (*p* < 0.05). (n = 4–6).

Disintegration time depended on both API type and compression pressure across all formulations. Figure 4 shows that, in high-solubility formulations, distinct pressure-dependent behaviors were observed, highlighting the role of API physicochemical properties and their interactions with the excipient matrix in governing disintegration performance.

Acetaminophen tablets exhibited rapid disintegration at all compression pressures, with times ranging from 0.84 ± 0.10 min at 296 MPa to 1.67 ± 0.29 min at 369 MPa, followed by a decrease at higher pressures to 1.13 ± 0.14 min at 591 MPa. This inverted U-shaped trend is consistent with the friability behavior observed for acetaminophen and reflects the brittle nature of the API.

In contrast, vitamin C tablets showed an increase in disintegration time with increasing compression pressure, from 2.11 ± 0.07 min at 296 MPa to 2.86 ± 0.05 min at 591 MPa, corresponding to a 1.4-fold increase (*p* < 0.001). This behavior is consistent with the increased densification of the tablet structure at higher compression pressures, which reduces tablet porosity and limits water penetration, thereby delaying tablet breakup. Additionally, the hydrophilic behavior of vitamin C may influence the hydration behavior of the starch-rich matrix during compaction, potentially contributing to the formation of a denser structure. However, specific intermolecular interactions were not directly measured in this study and would require further investigation to confirm this hypothesis.

**Figure 5:**
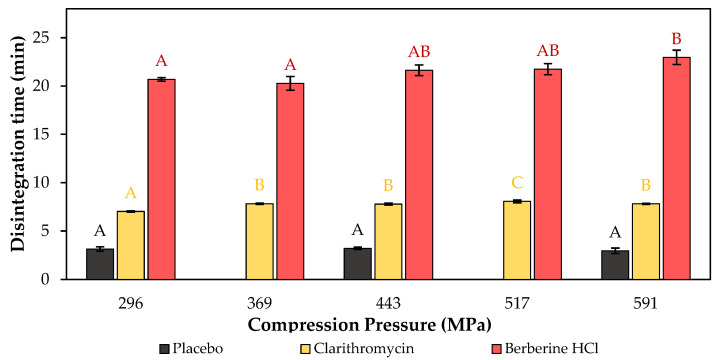
Effect of compression pressure on disintegration time for placebo and API-containing low-solubility formulations (clarithromycin (BCS Class II) and berberine HCl (BCS Class IV)) and Placebo using *Artocarpus altilis* as excipient. Statistical comparisons were performed across compression pressures within each formulation using one-way ANOVA and Tukey’s post hoc test (95% confidence level). Different letters indicate significant differences (*p* < 0.05). (n = 4–6).

For low-solubility formulations, as shown in Figure 5, disintegration times were prolonged and exhibited either a pressure-independent regime at higher compression levels (clarithromycin) or a progressive increase with compression pressure (berberine HCl).

Clarithromycin tablets exhibited significantly longer disintegration times, ranging from 7.02 ± 0.06 min to 8.1 ± 0.1 min. Beyond 369 MPa, no statistically significant changes were observed, indicating a pressure-independent regime at higher compression levels. This behavior is consistent with the limited aqueous solubility of clarithromycin, which restricts liquid penetration and delays matrix disruption.

Berberine HCl tablets exhibited the longest disintegration times, increasing from 20.70 ± 0.18 min at 296 MPa to 22.97 ± 0.74 min at 591 MPa across the compression range. Compared to placebo, berberine HCl tablets disintegrated approximately 7–8 times slower (*p* < 0.0001), showing the combined effects of low solubility and strong compact cohesion that collectively limit water penetration and tablet breakup.

These results are comparable to those previously reported for formulations containing extracted *A. altilis* starch as a pharmaceutical excipient. For example, metronidazole tablets prepared with *A. altilis* starch as a disintegrant showed hardness values of 49.6 to 56.5 N and disintegration times of 5.06 to 14.15 min [50], while the present study showed similar hardness values (1.05–1.5-fold higher) with shorter disintegration times (0.06–0.6-fold lower) for acetaminophen tablets. Similarly, formulations containing *A. altilis* starch as a disintegrant at different concentrations in acetaminophen tablets have been reported to produce tablets with hardness values of 12.5 to 14.2 N and disintegration times of 1.95 to 11.29 min [51], whereas the tablets evaluated here showed higher hardness (4.2- to 5.9-fold higher), but comparable disintegration behavior.

In addition, bilayer ibuprofen tablets containing modified *A. altilis* starch have been reported to exhibit friability values of 1.15 to 2.02% and disintegration times of 1.00 to 12.4 min [52], while clarithromycin tablets in the present study showed lower friability with comparable disintegration performance. Overall, despite differences in formulation composition and processing conditions, the tablet properties observed here fall within the ranges reported for *A. altilis* starch-based excipients in the literature. It should be noted that previous studies evaluated isolated or modified breadfruit starch, whereas the present study employed whole *A. altilis* powder; therefore, these comparisons should be interpreted as contextual benchmarks rather than direct formulation equivalence.

**Figure A2:**
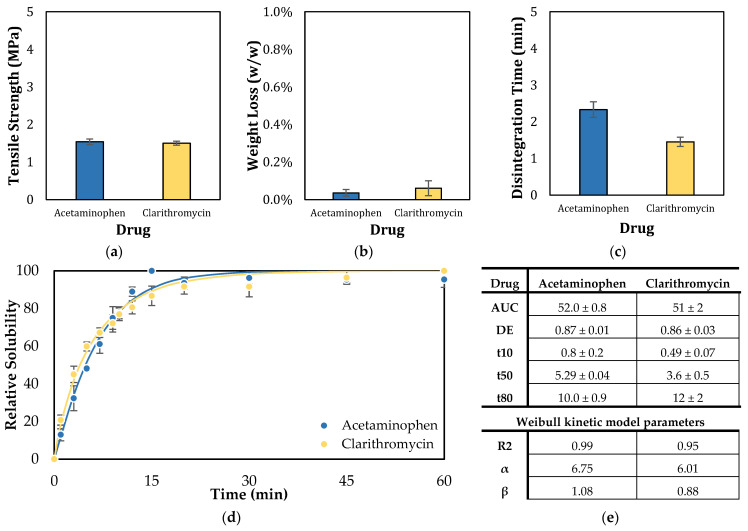
Performance attributes of commercial acetaminophen and clarithromycin tablets used as commercial formulations, including (**a**) tablet tensile strength, (**b**) friability, (**c**) disintegration time, (**d**) dissolution profiles, and (**e**) global dissolution performance metrics and Weibull kinetic model parameters.

**Figure A3:**
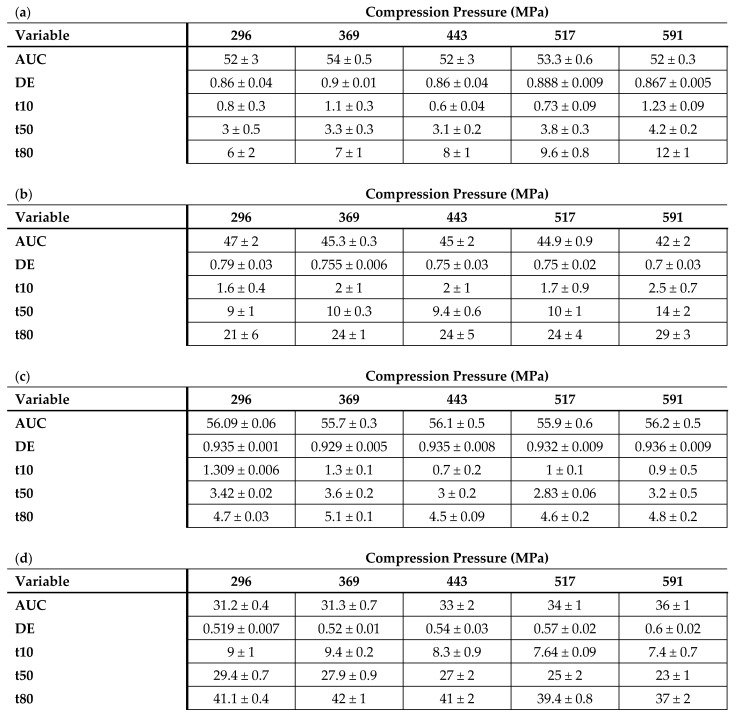
Dissolution performance descriptors as a function of compression pressure for all API-containing formulations. AUC represents the integrated area under the dissolution curve, whereas dissolution efficiency (DE) corresponds to the normalized AUC relative to complete dissolution over the same time interval (60 min). t10, t50, and t80 represent the time required to reach 10%, 50%, and 80% cumulative drug release, respectively. (**a**) Acetaminophen; (**b**) Clarithromycin; (**c**) Vitamin C; and (**d**) Berberine HCl. Values are reported as mean ± SD (n = 4–6).

The performance of commercial reference tablets for acetaminophen and clarithromycin is presented in Figure A2, showing comparative tensile strength, weight loss, and disintegration time values that serve as reference benchmarks for evaluating the mechanical strength and disintegration performance of the formulations developed in this work.

#### 3.3.3. Dissolution

**Figure 6:**
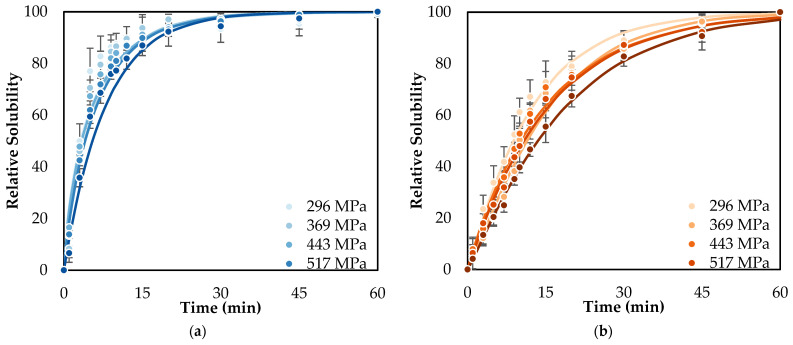

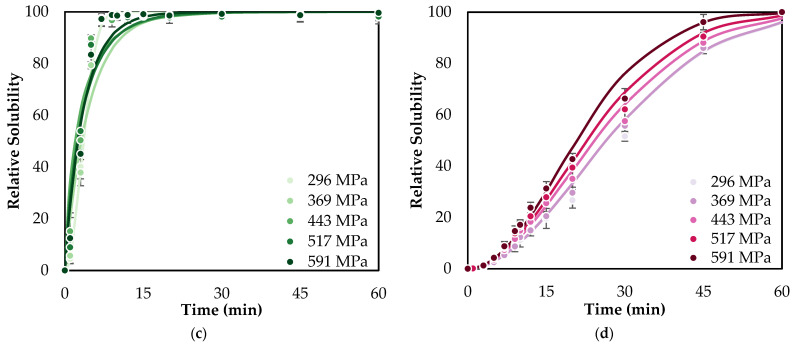
Effect of compression pressure on tablet relative solubility release profiles for API-containing formulations prepared with *Artocarpus altilis* as the excipient. (**a**) Acetaminophen; (**b**) clarithromycin; (**c**) vitamin C; and (**d**) berberine HCl. Symbols represent experimental dissolution data, and lines correspond to the Weibull kinetic model fitted to each formulation at the corresponding compression pressures. Color intensity represents the applied compression pressure, where lighter shades correspond to lower compression pressures and darker shades correspond to higher compression pressures. (n = 4–6).

Dissolution curves exhibited smooth, sigmoidal to asymptotic release behavior, as shown in Figure 6, indicating reproducible release kinetics and good agreement with the Weibull kinetic model. However, the degree of curve separation with compression pressure differed markedly across APIs, reflecting the interplay between API physicochemical properties and tablet compression conditions. The dissolution performance metrics and kinetic model parameters are provided in Figure A2 (Appendix B).

##### Acetaminophen

Acetaminophen formulations exhibited rapid dissolution (Figure 6a), characterized by a steep initial release followed by an early plateau. Regardless of compression pressure, tablets reached approximately 80% relative solubility within the first 10–15 min, and near-complete drug release was achieved before 30 min. Dissolution curves corresponding to different compression pressures were closely clustered, indicating limited sensitivity of acetaminophen dissolution to compaction pressure.

Dissolution efficiency remained constant across all compression pressures, ranging from 51.8 ± 2.6% at 296 MPa to 53.3 ± 0.6% at 517 MPa, indicating that overall release performance was not significantly affected by compaction pressure. Similarly, the AUC varied narrowly between 51.8 and 54.0, confirming comparable cumulative release across formulations.

**Figure A4:**
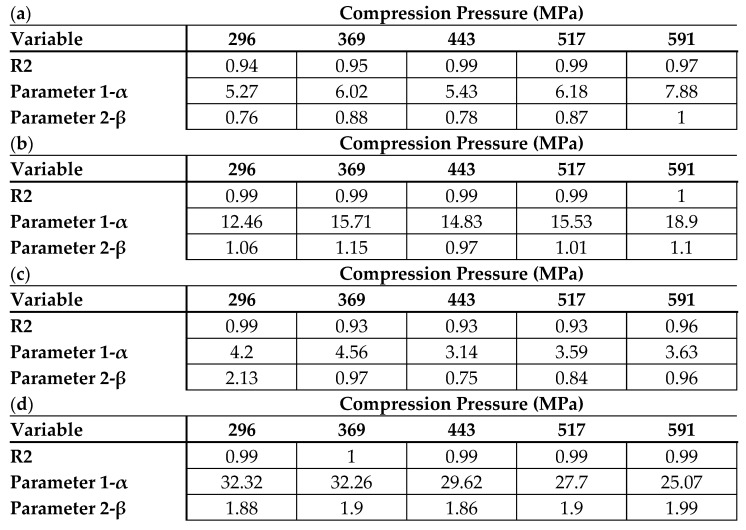
Weibull kinetic model parameters as a function of compression pressure for all API-containing formulations. (**a**) Acetaminophen; (**b**) Clarithromycin; (**c**) Vitamin C; and (**d**) Berberine HCl. Values are reported as mean ± SD (n = 4–6).

The Weibull model provided an excellent description of acetaminophen dissolution across all compression pressures (model parameters are shown in Figure A4a (Appendix C)). The shape parameter β remained between 0.76 and 1.00, indicating predominantly diffusion-controlled release kinetics, while the scale parameter α increased gradually with compression pressure, from 5.27 at 296 MPa to 7.88 at 591 MPa, consistent with the observed increase in characteristic dissolution times.

##### Clarithromycin

The dissolution profiles of clarithromycin tablets exhibited slower and more compression-sensitive dissolution behavior (Figure 6b), characterized by a gradual release phase and delayed approach to the asymptotic plateau, reaching approximately 80% of dissolution between 20.53 ± 5.59 min and 28.71 ± 3.31 min. A separation between dissolution curves was observed as a function of compression pressure, particularly in the intermediate time region. Tablets compressed at lower pressures exhibited faster release, while increasing compression pressure resulted in progressively slower dissolution kinetics, indicating that matrix densification significantly influenced drug release for this API.

Dissolution efficiency decreased with increasing compression pressure, from 47.26 ± 1.89% at 296 MPa to 42.26 ± 1.53% at 591 MPa, indicating a reduction in overall dissolution performance at higher compression levels. Similarly, the AUC decreased from 47.26 to 42.26, reflecting lower cumulative drug release over the dissolution period.

The Weibull model shape parameter β remained between 0.97 and 1.15, indicating near first-order release behavior (model parameters are shown in Figure A4b (Appendix C)). The scale parameter α increased with compression pressure, from 12.46 at 296 MPa to 18.90 at 591 MPa, showing a systematic increase in the characteristic dissolution time. Overall, clarithromycin tablets formulated with *Artocarpus altilis* exhibited pronounced sensitivity to compression pressure.

##### Vitamin C

The dissolution profiles of vitamin C tablets exhibited rapid dissolution, characterized by a steep initial release followed by an early plateau (Figure 6c). Regardless of compression pressure, tablets reached 80% relative solubility within the first 5–10 min, with near-complete drug release achieved shortly thereafter. The dissolution curves corresponding to different compression pressures completely overlapped throughout the entire dissolution period, indicating negligible sensitivity of vitamin C dissolution to compression pressure.

Dissolution efficiency remained constant across all compression pressures, with values of 0.93–0.94, indicating highly reproducible overall dissolution performance. Similarly, AUC values showed minimal variation, ranging from 55.7 to 56.2, confirming comparable cumulative release across formulations.

The Weibull model scale parameter α remained low (3.14–4.56), consistent with rapid dissolution kinetics, reflecting diffusion-dominated release with minimal structural constraint. The shape parameter β varied between 0.75 and 2.13. The model parameters are shown in Figure A4c (Appendix C).

##### Berberine HCl

The dissolution profiles of berberine HCl tablets exhibited slow and highly compression-dependent dissolution behavior, characterized by a prolonged initial phase followed by a gradual increase toward the asymptotic plateau. A systematic separation between dissolution curves was observed with increasing compression pressure across the entire time domain. Tablets compressed at higher pressures consistently exhibited faster release at similar time points. Although higher compression pressure typically reduces tablet porosity and may delay dissolution, the behavior observed in this formulation suggests a formulation-specific mechanism. This effect may reflect the balance between tablet structural integrity, disintegration behavior, and particle rearrangement within the starch-rich matrix during compaction, which can influence the exposure of the API to the dissolution medium.

Dissolution efficiency increased monotonically with compression pressure, from 31.15 ± 0.41% at 296 MPa to 36.01 ± 1.33% at 591 MPa, indicating a substantial improvement in overall dissolution performance with increasing compression pressure. A similar trend was observed for AUC, which increased from 31.15 to 36.01, reflecting enhanced cumulative drug release. While dissolution efficiency values for the other drug formulations remained relatively constant across compression pressures, the berberine HCl formulation exhibited a noticeable increase in DE, indicating greater sensitivity of dissolution performance to compression conditions. These results indicate that the influence of compression pressure on dissolution in this formulation likely reflects the balance between tablet consolidation and tablet breakup dynamics rather than a simple pressure–dissolution relationship.

The Weibull model shape parameter β remained consistently high, ranging from 1.86 to 1.99, indicating strongly sigmoidal release behavior with delayed onset followed by accelerated release, consistent with dissolution under solubility-limited conditions. The scale parameter α decreased systematically with compression pressure, from 32.32 at 296 MPa to 25.07 at 591 MPa, reflecting a reduction in the characteristic time required for drug release. The model parameters are shown in Figure A4d (Appendix C).

## 4. Conclusions

This study evaluated the tablet compression behavior of a starch-rich, natural-source excipient prepared from whole *Artocarpus altilis* material and assessed its performance when formulated with APIs representing all four BCS classes. By combining compression profile analysis with tablet performance testing, this work aimed to elucidate the mechanisms governing tablet formation and the functionality of the high starch excipient matrix in the presence of APIs with distinct physicochemical properties, particle size distributions, and morphologies.

The results showed that tabletability and compactability were the most sensitive descriptors for detecting API-dependent effects, whereas compressibility primarily reflected excipient-controlled densification. Differences in tensile strength development were associated with API particle size distribution and morphology, highlighting the importance of interparticle contact density and bonding efficiency in determining mechanical performance. These findings indicate that densification alone does not fully explain tablet strength; rather, the efficiency with which solid fraction is translated into tensile strength defines formulation robustness.

Tablet performance tests demonstrated that the *Artocarpus altilis* matrix maintains low friability while enabling rapid disintegration in highly soluble formulations and compression-dependent release control in poorly soluble systems. These results suggest that tablet performance reflects particle-level interactions between the API and the starch-rich matrix, in addition to compression pressure effects.

These findings indicate that tablet performance arises from formulation-dependent interactions between the API and the starch-rich matrix, while densification behavior remains primarily controlled by the excipient structure. The ability of *A. altilis*-based formulations to balance mechanical integrity and biopharmaceutical performance across diverse drug candidates highlights the potential of using sustainable and environmentally conscious formulations based on non-toxic natural-source excipients as multifunctional platforms for modern solid oral dosage forms.

## Data Availability

The original contributions presented in this study are included in the article. Further inquiries can be directed to the corresponding author.

## Abbreviations

The following abbreviations are used in this manuscript:

a	Compressibility coefficient
A	Compactability intercept
API	Active pharmaceutical ingredient
AUC	Area under the dissolution curve
b	Compressibility intercept
B	Tabletability intercept
BCS	Biopharmaceutics Classification System
D	Tablet diameter
D50	Volume-based median particle size
DE	Dissolution efficiency
F(t)	Fraction of drug dissolved at time t
FDA	U.S. Food and Drug Administration
h	Tablet thickness
k	Compactability coefficient
K	Tabletability coefficient
m	Tablet mass
MAR	Mean absolute residuals
P	Compression pressure
PSD	Particle size distribution
R^2^	Coefficient of determination
SSE	Sum of squared errors
t10	Time to 10% dissolution
t50	Time to 50% dissolution
t80	Time to 80% dissolution
USP	United States Pharmacopeia
V	Tablet volume
α	Weibull scale parameter
β	Weibull shape parameter
ρ_p_	particle true density
σₜ	Tablet tensile strength

## Appendix A

Appendix A presents the cumulative particle size distributions (PSD) of the excipient and API powders used in this study, as shown in Figure A1. The volumetric cumulative distributions provide a comparative assessment of particle size characteristics among placebo, acetaminophen, clarithromycin, vitamin C, and berberine HCl powders. Differences in median particle size (D50) and distribution breadth are visually evident and support the interpretation of compression behavior, compactability, and dissolution performance discussed in the main text.

## Appendix B

Appendix B summarizes the performance metrics obtained for the commercial tablet formulations used as controls in tensile strength, friability, disintegration, and dissolution testing, as seen in Figure A2. These data provide a benchmark for evaluating the performance of the *Artocarpus altilis*-based formulations discussed in the main text. Figure A2 reports the measured friability values, disintegration times, and global dissolution descriptors, including dissolution efficiency (DE) and area under the dissolution curve (AUC), for the corresponding commercial products at equivalent drug strengths.

## Appendix C

Appendix C presents the complete set of dissolution performance metrics and kinetic model parameters used to quantitatively support the dissolution behavior discussed in the main text. Figure A3 summarizes global dissolution descriptors, including area under the dissolution curve (AUC), dissolution efficiency (DE), and characteristic dissolution times (t_10_, t_50_, t_80_). Figure A4 reports the corresponding Weibull kinetic model parameters, including the scale (α) and shape (β) parameters, along with goodness-of-fit metrics.
